# The Ordering of Shannon Entropies for the Multivariate Distributions and Distributions of Eigenvalues

**DOI:** 10.3390/e21020201

**Published:** 2019-02-20

**Authors:** Ming-Tien Tsai, Feng-Ju Hsu, Chia-Hsuan Tsai

**Affiliations:** 1 Institute of Statistical Science, Academia Sinica, Taipei 11529, Taiwan; 2 Center for General Education, Saint Mary’s Junior College of Medicine, Nursing and Management, Yi-Lang 266, Taiwan; 3 Institute of Statistics, National Chiao-Tung University, Hsin-Chu 30010, Taiwan

**Keywords:** central Wishart and central MANOVA models, *MTP*_2_, noncentral Wishart and noncentral MANOVA models

## Abstract

In this paper, we prove the Shannon entropy inequalities for the multivariate distributions via the notion of convex ordering of two multivariate distributions. We further characterize the multivariate totally positive of order 2 ($MTP_{2}$) property of the distribution functions of eigenvalues of both central Wishart and central MANOVA models, and of both noncentral Wishart and noncentral MANOVA models under the general population covariance matrix set-up. These results can be directly applied to both the comparisons of two Shannon entropy measures and the power monotonicity problem for the MANOVA problem.

## 1. Introduction

Let $X_{1},X_{2},\cdots$ be independent random vectors with a common continuous distribution function F(x) which has density function $f\left(x\right),x\in R^{p}$ with respect to Lebesgue measure. The Shannon entropy for f(x) is denoted by

(1) $$\begin{array}{c} I_{S}\left(f\right)=\int_{-\infty }^{\infty }\left\{-\log f\left(x\right)\right\}f\left(x\right)dx. \end{array}$$

We further use F to denote all density functions whose Shannon entropies exist. That is

(2) $$\begin{array}{c} F=\{f:I_{S}\left(f\right)<\infty \}. \end{array}$$

Entropy inequality has been studied by several authors, such as Karlin and Rinott ([1,2], and references therein), who used the notions of majorization and Schur functions to study the Shannon (differential) entropy comparisons. Zografos and Nadarajah [3] pointed out that some comparisons of Shannon entropies can be induced by the affine transformation, those results show agreement on the concern that Shannon entropy is not scale invariant. Tsai [4] used the notion of convex ordering to investigate the Shannon inequalities for the univariate distributions. The novel finding of his approach is to adopt the difference of two Shannon entropy measures as a new measure, which is essentially symmetric. This new measure has some advantages over the well-known Kullback-Leibler divergence (relative entropy). The former one is designed for small or moderate deviation, however, the latter one is quite often used for large deviation. The measure of the difference between two Shannon entropies enjoys the finite sample property.

The key point of this method is to transform the convex ordering into the Lorenz ordering, which nicely depicts the stochastic ordering of two normalized distributions with the same starting and ending points, respectively. The presentation of the difference between two Shannon entropy measures can be represented in terms of a standard form of Kullback-Leibler divergence, however, it is interesting to note that the original Kullback-Leibler divergence does not have such kind of symmetric representation. Furthermore, the new measure is monotone likelihood ratio of two normalized density functions. This new measure provides us with some advantages for statistical inference.

The ordering of Shannon entropies can naturally occur when two underline distributions have the convex ordering or concave ordering relationship via the help of Lorenz ordering. In this paper, one of the main goals is to extend the result of Tsai [4] to the multivariate case, which is studied in Section 3. Please note that the notions of convex ordering of two distributions, monotone likelihood ratio (MLR) and totally positive of order 2 ($TP_{2}$) are essentially equivalent. We then adopt the notion of multivariate totally positive of order 2 ($MTP_{2}$) or multivariate reverse rule of order 2 ($MRR_{2}$) to get a more complete theory on Shannon entropy comparisons. Eigenvalues problem has become quite a hot topic at present. We further study the $MTP_{2}$ property of the distribution functions of eigenvalues of both central Wishart and central MANOVA models, and of both noncentral Wishart and noncentral MANOVA models under the general population covariance matrix set-up, respectively, in Section 4. The results can be directly applied to the comparisons of two Shannon entropy measures as well as the power monotonicity problem for the MANOVA problem, which has been open for a long time in the literature (Perlman and Olkin [5]). For a high-dimensional Wishart matrix with unknown scale times identity matrix as the population matrix, the $TP_{2}$ property of limiting empirical density function of eigenvalues is also studied in the Section 4.5. Mixture density functions, such as the noncentral chi-square density function and those presented in Section 4.3 and Section 4.4, play an important role in statistical inference. We also connected this notion with that of well-known Fisher’s multivariate analysis of variance in the final remark section.

## 2. The Univariate Distributions

Tsai [4] adopted the notion of convex ordering of distribution functions to study the orderings of Shannon entropy measures. For two univariate distributions F,G∈F, it is said that *F* is *c*-ordered (convex ordered) with respect to $G\;(F\leq_{c}G)$ if and only if $G^{-1}F$ is convex on the interval where 0<F(x)<1 (van Zwet [6]). The transformation of Barlow and Doksum [7] is to transform the convex ordering of distributions *F* and *G* to the stochastic ordering of distributions, it essentially can be viewed as a kind of Lorenz ordering (Gastwirth [8]).

Let $C_{F}\left(x\right)=G^{-1}F\left(x\right)$, then note that $dC_{F}\left(x\right)/dx=f(x)/g(G^{-1}F\left(x\right)),x\in \left(-\infty ,\infty \right)$. It can also be rewritten as $f(F^{-1}\left(u\right))/g(G^{-1}\left(u\right)),where\;u=F\left(x\right),u\in \left[0,1\right]$. We adopt the notion of Barlow and Doksum [7] to denote that $H_{F}\left(u\right)=\int_{0}^{G^{-1}\left(u\right)}f\left(F^{-1}G\left(t\right)\right)dt,u\in \left[0,1\right]$, which can be viewed as the inverse function of the transformation considered by Barlow and Doksum [7]. Please note that the notation in Barlow and Doksum [7] is originally defined that F(0)=0 and $F^{-1}\left(0\right)=0$, we use the general notations that F(−∞)=0 and $F^{-1}\left(0\right)=-\infty$ in this paper. Obviously, $H_{G}\left(u\right)=u,u\in \left[0,1\right]$. Under the assumption that $F\left(x\right)\leq_{c}G\left(y\right)$, Barlow and Doksum [7] pointed out that both $H_{F}\left(u\right)$ and $H_{G}\left(u\right),u\in \left[0,1\right]$ are two distribution functions. Let $h_{F}$ and $h_{G}$ be the corresponding density functions of $H_{F}$ and $H_{G}$, respectively, then we may see that $h_{F}\left(u\right)=f(F^{-1}\left(u\right))/g(G^{-1}\left(u\right))$ and $h_{G}\left(u\right)=1,u\in \left[0,1\right]$. Then, it is easy to note that the difference of two Shannon entropies can be represented as the Kullback-Leibler divergence of the uniform density function $h_{G}$ and the density function $h_{F}$.

Please note that if $F\left(x\right)\leq_{c}G\left(y\right)$, then $h_{F}\left(u\right)$ is nondecreasing in u∈[0,1], namely, it enjoys the property of monotone likelihood ratio. In addition, it gets across with the uniform density function $h_{G}\left(u\right)=1,u\in \left[0,1\right]$ at most once, the sign of the difference ($h_{F}\left(u\right)-h_{G}\left(u\right),u\in \left[0,1\right]$) of two density functions is from negative to nonnegative. Also note that $\int_{0}^{1}\left(h_{F}\left(v\right)-1\right)dv=0$, thus we have that $H_{F}\left(u\right)\leq u=H_{G}\left(u\right),u\in \left[0,1\right]$. Similarly, if $F\left(x\right)\leq_{c}G\left(y\right)$, then F(x)≤G(y), and $C_{F}\left(x\right)$ is a convex function in x,x∈(−∞,∞) (for the details see Shaked and Shanthikumar [9]). Namely, the convex ordering implies the stochastic ordering, however, the vice versa may not be true. We provide a simple counterexample for this. For 0≤x≤1, let $F\left(x\right)=x,G\left(x\right)={\left(3x-2\right)}^{3}/9+8/9$, then $F^{-1}\left(u\right)=u,G^{-1}\left(u\right)=\left({}^{3}\sqrt{9u-8}+2\right)/3,u\in \left[0,1\right]$. Thus, $f\left(F^{-1}\left(u\right)\right)=1,g\left(G^{-1}\left(u\right)\right)={\left(9u-8\right)}^{2/3}$ and $h_{F}\left(u\right)={\left(9u-8\right)}^{2/3}$. It is easy to see that F(u)≤G(u). However, $h_{F}\left(u\right)$ is not nondecreasing in *u*, u∈[0,1], that means that $F\left(x\right)\leq_{c}G\left(y\right)$ will not hold in this example. As such, we may conclude that if $F\left(x\right)\leq_{c}G\left(y\right)$, then F(x)≤G(y). However, if F(x)≤G(y), then $F\left(x\right)\leq_{c}G\left(y\right)$ may not hold generally.

**Theorem** **1** (Tsai [4])**.** *If $F\left(x\right)\leq_{c}G\left(y\right)$, then $I_{S}\left(f\right)\geq I_{S}\left(g\right)$.*

With the convex ordering assumption, i.e., $F\left(x\right)\leq_{c}G\left(y\right)$, the corresponding Lorenz ordering plays an important role for the proof of Theorem 1. To check whether the condition $F\left(x\right)\leq_{c}G\left(y\right)$ holds or not, one should make simultaneously check whether the condition F(x)≤G(y) holds or not. Similarly, define that $F\left(x\right)\leq_{concave}G\left(x\right)$ if and only if $F^{-1}G\left(x\right)$ is concave function in x,x∈(−∞,∞), then we have the following corollary.

**Corollary** **1.** *If $F\left(x\right)\leq_{concave}G\left(y\right)$, then $I_{S}\left(f\right)\leq I_{S}\left(g\right)$.*

**Proof.**  It can be easily followed by Theorem 1 via changing the sign of $logh_{F}\left(u\right)$. □

**Proposition** **1.** *Composition lemma (Karlin [10]). If K is $TP_{2}$ and L is $TP_{2}$ and ν is a σ-finite measure, then the convolution $M\left(x,y\right)=\int_{Z}K\left(x,z\right)L\left(z,y\right)d\nu \left(z\right)$ is $TP_{2}$.*

It can be proved similarly. We also note that if *K* is $TP_{2}$ and *L* is $RR_{2}$, then *M* is $RR_{2}$.

## 3. The Multivariate Distributions

By convex ordering transformation, we can neatly transform the difference of two Shannon entropy measures into the Lorenz ordering. In this section, we intend to extend this result to the multivariate case. It is natural to do so in the sense of conditional convex ordering for the multivariate case. Let $X={\left(X_{1},\ldots ,X_{p}\right)}^{⊤}$ and $Y={\left(Y_{1},\ldots ,Y_{p}\right)}^{⊤}$ be random vectors, and *f* and *g* be the density function of X and Y, respectively. Please note that the ${MTP}_{2}$ holds if and only if all the pairwise ${TP}_{2}$ hold (Fact 4.3.2 of Tong [11]), namely to examine the ${MTP}_{2}$ is the same as to examine all the pairwise ${TP}_{2}$ for any fixed pair. Hence without loss of generality, we may take p=2. Please note that $f\left(x_{1},x_{2}\right)=f_{1}\left(x_{1}\right)f_{2|1}\left(x_{2}|x_{1}\right)$, thus $f(x_{1},x_{2})$ is $TP_{2}$ if and only if $f_{1}\left(x_{1}\right)f_{2|1}\left(x_{2}|x_{1}\right)$ is $TP_{2}$. By the product property that the product function is $TP_{2}$ if two functions are each $TP_{2}$, respectively. Thus we may conclude that $f(x_{1},x_{2})$ is $TP_{2}$ if and only if both $f_{1}\left(x_{1}\right)$ and $f_{2|1}\left(x_{2}|x_{1}\right)$ are $TP_{2}$. That is to say that $F\left(x_{1},x_{2}\right)\leq_{c}G\left(y_{1},y_{2}\right)$ iff $F_{1}\left(x_{1}\right)\leq_{c}G_{1}\left(y_{1}\right)$ and $F_{2|1}\left(x_{2}|x_{1}\right)\leq_{c}G_{2|1}\left(y_{2}|y_{1}\right)$. Continue this process, for the general *p* case, we have the following.

**Proposition** **2.** *Let $c={\left(c_{1},\ldots ,c_{p}\right)}^{⊤}\in R^{p}$, then (i) $P\left\{Y_{1}>c_{1}\right\}\leq_{c}P\left\{X_{1}>c_{1}\right\}$, and (ii) $P\left\{Y_{j}>c_{j}|Y_{1}=y_{1},\ldots ,Y_{j-1}=y_{j-1}\right\}\leq_{c}P\left\{X_{j}>c_{j}|X_{1}=x_{1},\ldots ,X_{j-1}=x_{j-1}\right\}$ for all $x_{1}\leq y_{1},\ldots ,x_{j-1}\leq y_{j-1}$ and for all j=2,…,p if and only if $F\left(x\right)\leq_{c}G\left(y\right)$.*

From the results of Proposition 2, we are now in a position to use the convex ordering assumption for which $F_{j|1,\ldots ,j-1}\left(x_{j}|x_{1},\ldots ,x_{j-1}\right)\leq_{c}G_{j|1,\ldots ,j-1}\left(y_{j}|y_{1},\ldots ,y_{j-1}\right)$ to get each corresponding Lorenz ordering, ∀j=1,…,p, where j=1 denotes that $F_{1}\left(x_{1}\right)\leq_{c}G_{1}\left(y_{1}\right)$. With some elementary arguments, we then have the following.

**Theorem** **2.** *If $F\left(x\right)\leq_{c}G\left(y\right)$, then $I_{S}\left(f\right)\geq I_{S}\left(g\right)$.*

**Proof.**  For simplicity we may consider the situation when p=2 first. Let $g_{12}\left(y_{1},y_{2}\right)$ be the joint density function of random variables $Y_{1}$ and $Y_{2}$, $g_{2|1}\left(y_{2}|y_{1}\right)$ be the corresponding conditional density function of $Y_{2}$ given $Y_{1}=y_{1}$ and $G_{2|1}\left(y_{2}|y_{1}\right)$ be the corresponding distribution function. Let $f_{12}\left(x_{1},x_{2}\right),f_{2|1}\left(x_{2}|x_{1}\right)$ and $F_{2|1}\left(x_{2}|x_{1}\right)$ be defined similarly for the variables $X_{1}$ and $X_{2}$. Also let $U_{1}=F_{1}\left(x\right),U_{2}|u_{1}=F_{2|1}\left(x_{2}|x_{1}\right)\;\left(V_{1}=G_{1}\left(y\right),V_{2}|v_{1}=G_{2|1}\left(y_{2}|y_{1}\right)\right)$. Please note that

(3) $$\begin{array}{cc} I_{S}\left(f_{12}\right)-I_{S}\left(g_{12}\right) & =\int_{-\infty }^{\infty }\int_{-\infty }^{\infty }\left\{-\log f_{12}\left(x_{1},x_{2}\right)\right\}f_{12}\left(x_{1},x_{2}\right)dx_{1}dx_{2}\\ & \;\;\;-\int_{-\infty }^{\infty }\int_{-\infty }^{\infty }\left\{-\log g_{12}\left(y_{1},y_{2}\right)\right\}g_{12}\left(y_{1},y_{2}\right)dy_{1}dy_{2}\\ & =\int_{0}^{1}\int_{0}^{1}\left\{-\log\left[f_{1}\left(F_{1}^{-1}\left(u_{1}\right)\right)f_{2|1}\left(F_{2|1}^{-1}\left(u_{2}|u_{1}\right)\right)\right]\right\}du_{1}du_{2}\\ & \;\;\;-\int_{0}^{1}\int_{0}^{1}\left\{-\log\left[g_{1}\left(G_{1}^{-1}\left(v_{1}\right)\right)g_{2|1}\left(G_{2|1}^{-1}\left(v_{2}|v_{1}\right)\right)\right]\right\}dv_{1}dv_{2}\\ & =\int_{0}^{1}\int_{0}^{1}-\log\left[\frac{f_{1}\left(F_{1}^{-1}\left(u_{1}\right)\right)f_{2|1}\left(F_{2|1}^{-1}\left(u_{2}|u_{1}\right)\right)}{g_{1}\left(G_{1}^{-1}\left(u_{1}\right)\right)g_{2|1}\left(G_{2|1}^{-1}\left(u_{2}|u_{1}\right)\right)}\right]du_{1}du_{2}\\ & =\int_{0}^{1}-\log h_{F_{1}}\left(u_{1}\right)du_{1}+\int_{0}^{1}\left[\int_{0}^{1}-\log h_{F_{2|1}}\left(u_{2}|u_{1}\right)du_{2}\right]du_{1}, \end{array}$$

where $h_{F_{1}}\left(u_{1}\right)=f_{1}\left(F_{1}^{-1}\left(u_{1}\right)\right)/g_{1}\left(G_{1}^{-1}\left(u_{1}\right)\right),h_{G_{1}}\left(u_{1}\right)=1$ and $h_{F_{2|1}}\left(u_{2}|u_{1}\right)=f_{2|1}\left(F_{2|1}^{-1}\left(u_{2}|u_{1}\right)\right)/g_{2|1}\left(G_{2|1}^{-1}\left(u_{2}|u_{1}\right)\right),h_{G_{2|1}}\left(u_{2}|u_{1}\right)=1$. Furthermore, by Proposition 2, we may note that $F\left(x_{1}\right)\leq_{c}G\left(y_{1}\right)$, and thus via the arguments in Section 2, we have that $h_{F_{1}}\left(u_{1}\right),u_{1}\in \left[0,1\right]$ is the density function. Similarly, we also have that $F_{2|1}\left(x_{2}|x_{1}\right)\leq_{c}G_{2|1}\left(y_{2}|y_{1}\right)$, and then $h_{F_{2|1}}\left(u_{2}|u_{1}\right),u_{2}\in \left[0,1\right]$ is the density function. Thus by the information inequality, the first term of the right hand side of Equation (3) is non-negative and the second term insight bracket is non-negative too, and hence the second term of the right hand side of Equation (3) is non-negative. Thus we may conclude that $I_{S}\left(f_{12}\right)\geq I_{S}\left(g_{12}\right)$.

Let $U_{i}|u_{1},\ldots ,u_{i-1}=F_{i|1,\ldots ,i-1}\left(x_{i}|x_{1},\ldots ,x_{i-1}\right)$, $V_{i}|v_{1},\ldots ,v_{i-1}=G_{i|1,\ldots ,i-1}\left(y_{i}|y_{1},\ldots ,i_{p-1}\right)$ and $h_{F_{i|1,\ldots ,i-1}}\left(u_{i}|u_{1},\ldots ,u_{i-1}\right)=f_{i|1,\ldots ,i-1}\left(F_{i|1,\ldots ,i-1}^{-1}\left(u_{i}|u_{1},\ldots ,u_{i-1}\right)\right)/g_{i|1,\ldots ,i-1}\left(G_{i|1,\ldots ,i-1}^{-1}\left(v_{i}|v_{1},\ldots ,v_{i-1}\right)\right),i=2,\ldots ,p$. Continuing the processes, finally for general *p* we have

(4) $$\begin{array}{cc} I_{S}\left(f\right)-I_{S}\left(g\right) & =\int_{-\infty }^{\infty }\left\{-\log f\left(x\right)\right\}f\left(x\right)dx-\int_{-\infty }^{\infty }\left\{-\log g\left(y\right)\right\}g\left(y\right)dy\\ & =\int_{0}^{1}-\log h_{F_{1}}\left(u_{1}\right)du_{1}+\int_{0}^{1}\left[\int_{0}^{1}-\log h_{F_{2|1}}\left(u_{2}|u_{1}\right)du_{2}\right]du_{1}\\ & \;\;\;+\ldots +\int_{0}^{1}\ldots \int_{0}^{1}\left[\int_{0}^{1}-\log h_{F_{p|1,\ldots ,p-1}}\left(u_{p}|u_{1},\ldots ,u_{p-1}\right)du_{p}\right]du_{p-1}\ldots du_{1}\\ & \geq 0. \end{array}$$

Hence, the theorem follows. □

For two arbitrary densities $f_{g},f_{h}$, the Kullback-Leibler information discrimination is defined by $K\left(f_{g},f_{h}\right)=\int_{-\infty }^{\infty }f_{g}\left(x\right)\log\left\{f_{g}\left(x\right)/f_{h}\left(x\right)\right\}dx$. It is non-negative for all $f_{g},f_{h}$, and is equal to zero if $f_{g}=f_{h}$ almost everywhere. Let E[X] denote the expectation of random variable *X*. If $F\left(x\right)\leq_{c}G\left(y\right)$, from Equation (4) we have the following representation:

(5) $$\begin{array}{cc} I_{S}\left(f\right)-I_{S}\left(g\right) & =K\left(1,h_{F_{1}}\right)+E_{U_{1}}\left[K\left(1,h_{F_{2|1}}\right)\right]+\ldots +E_{U_{1},\ldots ,U_{p-1}}\left[K\left(1,h_{F_{p|1,\ldots ,p-1}}\right)\right], \end{array}$$

where $E_{U_{1},\ldots ,U_{i-1}}$ denotes the expectation of independent uniform densities of $\left(U_{1},\ldots ,U_{i-1}\right)$, $U_{i}\in \left[0,1\right],i=1,\ldots ,p-1$.

**Corollary** **2.** *If $F\left(x\right)\leq_{concave}G\left(y\right)$, then $I_{S}\left(f\right)\leq I_{S}\left(g\right)$.*

As we have seen, the proof of Theorem 2 is surprisingly easy. However, to characterize the property of convex ordering for two multivariate distributions is sometimes much more complicated via the conditional approach than to characterize the $MTP_{2}$ property directly. The convex ordering of two multivariate distributions is essentially equivalent to the notion of $MTP_{2}$, so does the concave ordering to the $MRR_{2}$. Karlin and Rinott ([12,13]) had studied several $MTP_{2}$ and $MRR_{2}$ distributions, respectively. Some other often seen models related to Theorem 2 and Corollary 2 are exampled in the following.

**Example** **1.** *Let y=Ax, where A is a nonsingular matrix. Then $I_{S}\left(g\right)=log\left|A\right|+I_{S}\left(f\right)$, where |A| denotes the determinant of matrix A (Zografos and Nadarajah [3]). Thus we have $I_{S}\left(f\right)\geq I_{S}\left(g\right)$ iff |A|≤1.*

**Example** **2.** *Let $B^{p}=\left\{{\left(x_{1},\ldots ,x_{p}\right)}^{⊤}:x_{i}>0,i=1,\ldots ,p,\sum_{i=1}^{p}x_{i}\leq 1\right\}$ be the p-dimensional simplex, and let*

$$\begin{array}{c} f\left(x,a_{1},\ldots ,a_{p};b_{1},\ldots ,b_{p}\right)=c\prod_{i=1}^{p}x_{i}^{a_{i}-1}{\left(1-\sum_{k=1}^{i}x_{k}\right)}^{b_{i}-1} \end{array}$$

*be the generalized Dirichlet density function with parameters, $a_{i}>0,b_{i}>0,i=1,\cdots ,p$ and*

$$\begin{array}{c} c=\prod_{i=1}^{p}\frac{\Gamma (\sum_{k=i}^{p}\left(a_{k}+b_{k}-1\right)+1)}{\Gamma \left(a_{i}\right)\Gamma \left(b_{i}+\sum_{k=i}^{p}\left(a_{k}+b_{k}-1\right)\right)}. \end{array}$$

*It is easy to note that $x_{i}^{a_{i}-1}$ is $TP_{2}$ in $\left(x_{i},a_{i}\right)$, ${\left(1-\sum_{k=1}^{i}x_{k}\right)}^{b_{i}-1}$ is $RR_{2}$ in $\left(x_{i},b_{i}\right)$ and ${\left(1-\sum_{k=1}^{i}x_{k}\right)}^{b_{i}-1}$ is pairwisely $RR_{2}$ in $\left(x_{i},x_{j}\right)$ when $b_{i}\geq 1$. Then, by Proposition 2, we have (i) $\prod_{i=1}^{p}x_{i}^{a_{i}-1}$ is $MTP_{2}$ and (ii) $\prod_{i=1}^{p}{\left(1-\sum_{k=1}^{i}x_{k}\right)}^{b_{i}-1}$ is $MRR_{2}$ when $b_{i}\geq 1,\forall i=1,\ldots ,p$. Thus, we may conclude that the generalized Dirichlet density function is $MRR_{2}$ when $b_{i}\geq 1,\forall i=1,\ldots ,p$. In addition, Corollary 2 is applicable.*

**Example** **3.** *Gupta and Richards [14] considered the multivariate Liouville distribution which the density function is of the form*

$$\begin{array}{c} f\left(x,\theta \right)=c\left(\theta \right)g\left(\sum_{i=1}^{p}x_{i}\right)\prod_{i=1}^{p}x_{i}^{\theta_{i}}, \end{array}$$

*where $g:R^{+}\to R^{+}$ is continuous, and $\theta ,x\in R^{+p}$ and*

$$\begin{array}{c} c^{-1}\left(\theta \right)=\frac{\prod_{i=1}^{p}\Gamma \left(\theta_{i}\right)}{\Gamma (\sum_{i=1}^{p}\theta_{i})}\int_{0}^{\infty }t^{\sum_{i=1}^{p}\theta_{i}-1}g\left(t\right)dt. \end{array}$$

*Please note that $\prod_{i=1}^{p}x_{i}^{\theta_{i}}$ is $MTP_{2}$. By Proposition 2, it is easy to see that f(x,θ) is $MTP_{2}$ ($MRR_{2}$) if and only if $g(x_{1}+x_{2})$ is $TP_{2}\;\left(RR_{2}\right)$ in $\left(x_{1},x_{2}\right)$ on $R^{+2}$. The $MRR_{2}$ property of some special cases of multivariate Liouville distributions, such as Dirichlet distributions and inverted Dirichlet distributions, are studied by Karlin and Rinott [13].*

**Example** **4.** *In biostatistics, many studies are under the setup of well-known Cox proportional hazard model (Cox [15]), and its basic model is assumed to be that $\bar{G}\left(x\right)={\left(\bar{F}\left(x\right)\right)}^{\lambda },\lambda >0$, where $\bar{G}\left(x\right)=1-G\left(x\right)$. Without loss of generality, we may assume p=2. Write $\bar{G}\left(x\right)={\bar{G}}_{1}\left(x_{1}\right){\bar{G}}_{2|1}\left(x_{2}|x_{1}\right)$ and $\bar{F}\left(x\right)={\bar{F}}_{1}\left(x_{1}\right){\bar{F}}_{2|1}\left(x_{2}|x_{1}\right)$. Let $G_{1}\left(x\right)=u_{1},u_{1}\in \left[0,1\right]$, then we have $F_{1}\left(G_{1}^{-1}\left(u_{1}\right)\right)=1-{\left(1-u_{1}\right)}^{1/\lambda }$, thus we have $dF_{1}\left(G_{1}^{-1}\left(u_{1}\right)\right)/du_{1}={\left(1-u_{1}\right)}^{\left(1/\lambda -1\right)}/\lambda$, which is nondecreasing in $u_{1}$ if λ≥1. As such, we have $G_{1}^{-1}\left(u_{1}\right)\leq_{c}F_{1}^{-1}\left(u_{1}\right),u_{1}\in \left[0,1\right]$ when λ≥1. Namely, when λ≥1 we have $F_{1}\left(x_{1}\right)\leq_{c}G_{1}\left(x_{1}\right)$. Let $G_{2|1}\left(x_{2}|x_{1}\right)=u_{2}$, similar arguments as above, we may have that $F_{2|1}\left(x_{2}|x_{1}\right)\leq_{c}G_{2|1}\left(x_{2}|x_{1}\right)$ when λ≥1. Thus, by Proposition 2, we may have that $F\left(x\right)\leq_{c}G\left(x\right)$ when λ≥1. This procedure can be directly generalized to the general p case by mathematical induction. As such, for the positive disease gene case λ≤1, then by Theorem 2 we may conclude that $I_{S}\left(f\right)\leq I_{S}\left(g\right)$. Similarly, for the negative disease gene case λ≥1, then $I_{S}\left(f\right)\geq I_{S}\left(g\right)$.*

**Example** **5.** *The density function of eigenvalues for a multivariate beta matrix is of the form*

$$\begin{array}{c} f\left(x\right)=c\prod_{i=1}^{p}x_{i}^{\frac{1}{2}\left(n_{1}-p+1\right)-1}{\left(1-x_{i}\right)}^{\frac{1}{2}\left(n_{2}-p+1\right)-1}\prod_{1\leq i<j\leq p}\left|x_{i}-x_{j}\right|, \end{array}$$

*where $0<x_{i}<1,i=1,\ldots ,p,n_{1},n_{2}>p$, and*

$$\begin{array}{c} c=\prod_{i=1}^{p}\frac{\Gamma \left(\frac{3}{2}\right)\Gamma \left(\frac{1}{2}\left(n_{1}+n_{2}-p+i\right)\right)}{\Gamma \left(1+\frac{i}{2}\right)\Gamma \left(\frac{1}{2}\left(n_{1}-p+i\right)\right)\Gamma \left(\frac{1}{2}\left(n_{1}-p+i\right)\right)} \end{array}$$

*(Dumitriu [16]; Peddada and Richards [17]). Please note that the Vandermonde determinant $\prod_{1\leq i<j\leq p}\left|x_{i}-x_{j}\right|$ is $MTP_{2}$, for the details see Dykstra and Hewett [18]. Using Proposition 2, it is easy to see that $\prod_{i=1}^{p}x_{i}^{\left(n_{1}-p+1\right)/2-1}$ is $TP_{2}$ in ($x_{i},n_{1}-p+1$), and $\prod_{i=1}^{p}{\left(1-x_{i}\right)}^{\left(n_{2}-p+1\right)/2-1}$ is $RR_{2}$ in ($x_{i},n_{2}-p+1$). Thus, $\prod_{i=1}^{p}x_{i}^{(n_{1}-p+1)/2-1}{\left(1-x_{i}\right)}^{(n_{2}-p+1)/2-1}$ is $MRR_{2}$. Then, we may conclude that the density function, which is the product of these functions, is $MRR_{2}$. In addition, Corollary 2 is applicable.*

## 4. The Central Wishart and Central MANOVA Models, and Noncentral Wishart and Noncentral MANOVA Models

Theorem 2 states that if the density function has the $MTP_{2}$ property, then the corresponding ordering of Shannon entropies holds, namely the $MTP_{2}$ (or $MRR_{2})$ property of density function ensures the ordering of Shannon entropies. The $MTP_{2}$ also implies the power monotonicity property of MANOVA tests, for which based on the monotone function of eigenvalues. Eigenvalues play an important role in statistical inference. Example 5 deals with the density function of eigenvalues of MANOVA models (or Jacobi ensembles) when the population covariance matrix is assumed to be an identity matrix (Σ=I). Two other often seen models are (i) Gaussian (or Hermite) ensembles,

$$\begin{array}{c} f\left(x\right)={\left(2\pi \right)}^{-\frac{p}{2}}\prod_{i=1}^{p}\frac{\Gamma (\frac{3}{2})}{\Gamma (1+\frac{i}{2})}e^{-\frac{1}{2}\sum_{1}^{p}x_{i}^{2}}\prod_{1\leq i<j\leq p}\left|x_{i}-x_{j}\right|, \end{array}$$

and (ii) Wishart models (or Laguerre ensembles),

$$\begin{array}{c} f\left(x\right)=2^{-\frac{p(p+1)}{2}}\prod_{i=1}^{p}\frac{\Gamma (\frac{3}{2})}{\Gamma \left(1+\frac{i}{2}\right)\Gamma \left(\frac{1}{2}\left(n_{1}-p+1\right)\right)}\prod_{i=1}^{p}x_{i}^{\frac{1}{2}\left(n_{1}-p+1\right)-1}e^{-\frac{1}{2}\sum_{1}^{p}x_{i}}\prod_{1\leq i<j\leq p}\left|x_{i}-x_{j}\right| \end{array}$$

(Dumitriu [16]). From similar arguments to Example 5, it is easy to see that the density function f(x) is $MTP_{2}$ either for (i) (the identity population covariance matrix is an *M*-matrix) or for (ii) (gamma type density function), respectively, we omit the details.

For statistical inference, generally the population covariance matrix is unknown. In this section, we further study the $MTP_{2}$ property of the distribution functions of eigenvalues of central Wishart and MANOVA models, and of noncentral Wishart and MANOVA models (James [19]; Muirhead [20]) under the general population covariance matrix set-up.

### 4.1. Type ${}_{0}F_{0}$, Exponential: Eigenvalues of a Wishart Matrix

Suppose that the columns of a matrix p×n matrix X are independently normally distributed with covariance matrix Σ and E(X)=0. Let $L=\mathrm{diag}(l_{1},\ldots ,l_{p})$, where $l_{i}$ be the *i*th largest eigenvalue of $XX^{⊤}$. Similarly, let $\Delta =\mathrm{diag}(\delta_{1},\ldots ,\delta_{1})$, where $\delta_{i}$ be the *i*th largest eigenvalue of Σ. Write $\Sigma =Q\Delta Q^{⊤}$ and $XX^{⊤}=ULU^{{}^{\prime }}$, where Q,U∈O(p) with O(p) being the group of p×p orthogonal matrices. Also denote (dU) the *m*-form of U with $m=\frac{1}{2}p\left(p+1\right)$ and tr denotes the trace operator, then

(6) $$\begin{array}{cc} {}_{0}F_{0}\left(-\frac{1}{2}\Sigma^{-1},XX^{⊤}\right) & =\int_{O(p)}e^{-\frac{1}{2}\mathrm{tr}\left(Q\Delta^{-1}Q^{⊤}ULU^{⊤}\right)}\left(dU\right)\\ & =\int_{O(p)}e^{-\frac{1}{2}\mathrm{tr}\left(\Delta^{-1}HLH^{⊤}\right)}\left(dH\right)\\ & ={}_{0}F_{0}\left(-\frac{1}{2}\Delta^{-1},L\right), \end{array}$$

where $H=Q^{⊤}U\in O(p)$ and noting that (dU)=(dH). Due to the invariant property, the density function of L depends only upon Δ, which is of the form

(7) $$\begin{array}{c} \phi_{\delta }\left(l_{1},\ldots ,l_{p}\right)={\left|\Delta \right|}^{-\frac{1}{2}n}{}_{0}F_{0}\left(-\frac{1}{2}\Delta^{-1},L\right)\phi_{0}\left(l_{1},\ldots ,l_{p}\right), \end{array}$$

where

(8) $$\begin{array}{c} \phi_{0}\left(l_{1},\ldots ,l_{p}\right)=\frac{\pi^{\frac{1}{2}p^{2}}}{2^{\frac{1}{2}pn}\Gamma_{p}\left(\frac{1}{2}n\right)\Gamma_{p}\left(\frac{1}{2}p\right)}{\left|L\right|}^{\frac{1}{2}\left(n-p-1\right)}\prod_{i<j}\left(l_{i}-l_{j}\right) \end{array}$$

with $\Gamma_{p}\left(a\right)=\pi^{\frac{1}{4}p\left(p-1\right)}\sum_{i=1}^{p}\Gamma \left(a-\frac{1}{2}\left(i-1\right)\right)$.

**Theorem** **3.** *${}_{0}F_{0}\left(-\frac{1}{2}\Delta^{-1},L\right)$ is $MTP_{2}$.*

**Proof.**  Please note that

$$\begin{array}{cc} \frac{\partial \log{}_{0}F_{0}\left(-\frac{1}{2}\Delta^{-1},L\right)}{\partial L} & =\frac{\int_{O(p)}e^{-\frac{1}{2}\mathrm{tr}\left(\Delta^{-1}HLH^{⊤}\right)}\left(-\frac{1}{2}\Delta^{-1}HH^{⊤}\right)\left(dH\right)}{\int_{O(p)}e^{-\frac{1}{2}\mathrm{tr}\left(\Delta^{-1}HLH^{⊤}\right)}\left(dH\right)}\\ & =\frac{\int_{O(p)}e^{-\frac{1}{2}\mathrm{tr}\left(\Delta^{-1}HLH^{⊤}\right)}\left(-\frac{1}{2}\Delta^{-1}\right)\left(dH\right)}{\int_{O(p)}e^{-\frac{1}{2}\mathrm{tr}\left(\Delta^{-1}HLH^{⊤}\right)}\left(dH\right)}\\ & =-\frac{1}{2}\Delta^{-1}. \end{array}$$

Thus, it is easy to see that

$$\begin{array}{c} \frac{\partial^{2}\log{}_{0}F_{0}\left(-\frac{1}{2}\Delta^{-1},L\right)}{\partial L^{{}^{\prime }}\partial L}=0 \end{array}$$

and

$$\begin{array}{c} \frac{\partial^{2}\log{}_{0}F_{0}\left(-\frac{1}{2}\Delta^{-1},L\right)}{\partial \Delta \partial L}=\frac{1}{2}\Delta^{-2}, \end{array}$$

respectively. Please note that Δ is a positive diagonal matrix, which is clearly positive definite. Hence the theorem follows. □

By the result of Dykstra and Hewett [18], it is easy to note that $\phi_{0}\left(l_{1},\ldots ,l_{p}\right)$ enjoys the $MTP_{2}$ property. Hence, by Theorem 3 we may conclude that the density function $\phi_{\delta }\left(l_{1},\ldots ,l_{p}\right)$ of eigenvalues of $XX^{⊤}$, which is the product of two $MTP_{2}$ functions (Karlin and Rinott [12]), is $MTP_{2}$.

### 4.2. Type ${}_{1}F_{0}$, Binomial Series: Eigenvalues When $\Sigma_{1}\neq \Sigma_{2}$

Suppose that the $p\times n_{1}$ matrix variate X with $n_{1}$ independent columns distributed as $N(0,\Sigma_{1})$ and $p\times n_{2}$ matrix variate Y with $n_{2}$ independent columns distributed as $N(0,\Sigma_{2})$. Also let $F=\mathrm{diag}(f_{1},\ldots ,f_{p})$ and $\Omega =\mathrm{diag}(\omega_{1},\ldots ,\omega_{p})$, where $f_{i}$ is the *i*th largest eigenvalue of

(9) $$\begin{array}{c} |XX^{⊤}-fYY^{⊤}|=0, \end{array}$$

and

(10) $$\begin{array}{c} |\Sigma_{1}-\omega \Sigma_{2}|=0. \end{array}$$

Let

(11) $$\begin{array}{c} {}_{1}F_{0}\left(a;-S,T\right)=\int_{O(p)}{\left|I+SHTH^{⊤}\right|}^{-a}\left(dH\right), \end{array}$$

the equation is well defined for all S and T being positive definite. Without loss of generality, we may take both S and T are positive diagonal matrices. Then the joint density function of eigenvalues $f_{1},\ldots ,f_{p}$ is of the form

(12) $$\begin{array}{c} \phi_{\omega }\left(f_{1},\cdots ,f_{p}\right)={\left|\Omega \right|}^{-\frac{1}{2}n_{1}}{}_{1}F_{0}\left(\frac{1}{2}\left(n_{1}+n_{2}\right);-\Omega^{-1},F\right)\phi_{*}\left(f_{1},\ldots ,f_{p}\right), \end{array}$$

where

(13) $$\begin{array}{c} \phi_{*}\left(f_{1},\ldots ,f_{p}\right)=\frac{\pi^{\frac{1}{2}p^{2}}\Gamma_{p}\left(\frac{1}{2}\left(n_{1}+n_{2}\right)\right)}{\Gamma_{p}\left(\frac{1}{2}n_{1}\right)\Gamma_{p}\left(\frac{1}{2}n_{2}\right)\Gamma_{p}\left(\frac{1}{2}p\right)}{\left|F\right|}^{\frac{1}{2}\left(n_{1}-p-1\right)}\prod_{i<j}\left(f_{i}-f_{j}\right). \end{array}$$

**Theorem** **4.** *${}_{1}F_{0}\left(\frac{1}{2}n;-\Omega^{-1},F\right)$ is $MTP_{2}$, where $n=n_{1}+n_{2}$.*

**Proof.**  Please note that ${}_{1}F_{0}\left(\frac{1}{2}n;-\Omega^{-1},F\right)=\int_{O(p)}|I+\Omega^{-1}HFH^{⊤}{|}^{-n/2}\left(dH\right)={\left|\Omega \right|}^{n/2}\int_{O(p)}{\left|\Omega +HFH^{⊤}\right|}^{-n/2}\left(dH\right)$, thus

$$\begin{array}{cc} \frac{\partial \log{}_{1}F_{0}\left(\frac{1}{2}n;-\Omega^{-1},F\right)}{\partial F} & =\frac{\int_{O(p)}-\frac{1}{2}n{\left|\Omega +HFH^{⊤}\right|}^{-\frac{1}{2}n}{\left(\Omega +HFH^{⊤}\right)}^{-1}\left(dH\right)}{\int_{O(p)}{\left|\Omega +HFH^{⊤}\right|}^{-\frac{1}{2}n}\left(dH\right)}. \end{array}$$

Write $B(\Omega ,F)=\int_{O(p)}{\left|\Omega +HFH^{⊤}\right|}^{-n/2}\left(dH\right)>0$, then after some calculations

$$\begin{array}{cc} \frac{\partial^{2}\log{}_{1}F_{0}\left(\frac{1}{2}n;-\Omega^{-1},F\right)}{\partial \Omega \partial F} & =\{\frac{1}{4}n^{2}\int_{O(p)}|\Omega +HFH^{⊤}{|}^{-\frac{1}{2}n}{\left(\Omega +HFH^{⊤}\right)}^{-2}\left(dH\right)\times B\left(\Omega ,F\right)\\ & -\frac{1}{4}n^{2}[\int_{O(p)}|\Omega +HFH^{⊤}{{|}^{-\frac{1}{2}n}{\left(\Omega +HFH^{⊤}\right)}^{-1}\left(dH\right)]}^{2}\\ & +\frac{1}{2}n\int_{O(p)}|\Omega +HFH^{⊤}{|}^{-\frac{1}{2}n}{\left(\Omega +HFH^{⊤}\right)}^{-2}\left(dH\right)\times B\left(\Omega ,F\right)\}/B^{2}\left(\Omega .F\right) \end{array}$$

By Schwartz inequality, we then have

$$\begin{array}{cc} \frac{\partial^{2}\log{}_{1}F_{0}\left(\frac{1}{2}n;-\Omega^{-1},F\right)}{\partial \Omega \partial F} & \geq \frac{\frac{1}{2}n\int_{O(p)}{\left|\Omega +HFH^{⊤}\right|}^{-\frac{1}{2}n}{\left(\Omega +HFH^{⊤}\right)}^{-2}\left(dH\right)}{B(\Omega ,F)}\\ & =\frac{1}{2}nE_{f}\left[{\left(\Omega +HFH^{⊤}\right)}^{-2}|F;\Omega \right]\\ & \geq 0 \end{array}$$

with probability one, where $f\left(H|F;\Omega \right)=|\Omega +HFH^{⊤}{|}^{-n/2}/B(\Omega ,F)$ can be viewed as the conditional density function of H given F. Please note that the matrix $HFH^{⊤}$ is positive definite with probability one, thus the conditional expectation $E_{f}\left[{\left(\Omega +HFH^{⊤}\right)}^{-2}|F;\Omega \right]$ is positive definite with probability one. Hence, the theorem follows. □

Please note that $\phi_{*}\left(f_{1},\ldots ,f_{p}\right)$ is $MTP_{2}$. Thus, by Theorem 4 we have that the density function $\phi_{\omega }\left(f_{1},\ldots ,f_{p}\right)$ is $MTP_{2}$.

### 4.3. Type ${}_{0}F_{1}$, Bessel: Noncentral Means with Known Covariance

Suppose that the columns of a matrix p×n matrix X are independently normally distributed with covariance matrix Σ and E(X)=M. Let $w_{i}$ be the eigenvalues of $|XX^{⊤}-w\Sigma |=0$ and $W=\mathrm{diag}(w_{1},\ldots ,w_{p})$, then its density function depends only upon $\Omega =\mathrm{diag}(\omega_{1},\ldots ,\omega_{p})$, where $\omega_{i}$ are the eigenvalues of $|MM^{⊤}-\omega \Sigma |=0$, and is

(14) $$\begin{array}{cc} \psi_{\omega }\left(w_{1},\ldots ,w_{p};\Omega \right)=e^{-\frac{1}{2}\mathrm{tr}\Omega }{}_{0}F_{1}(\frac{1}{2}n; & \frac{1}{4}\Omega ,W)\psi_{0}\left(w_{1},\ldots ,w_{p}\right), \end{array}$$

where

(15) $$\begin{array}{c} \psi_{0}\left(w_{1},\cdots ,w_{p}\right)=\frac{\pi^{\frac{1}{2}p^{2}}}{2^{\frac{1}{2}pn}\Gamma_{p}\left(\frac{1}{2}n\right)\Gamma_{p}\left(\frac{1}{2}p\right)}e^{-\frac{1}{2}\mathrm{trw}}{\left|W\right|}^{\frac{1}{2}\left(n-p-1\right)}\prod_{i<j}\left(w_{i}-w_{j}\right). \end{array}$$

Furthermore, from Equation (32) of James [19], we have the inverse Laplace transform

(16) $$\begin{array}{cc} {}_{0}F_{1}\left(\frac{1}{2}n;\Omega ,W\right) & =\frac{2^{\frac{1}{2}p\left(p-1\right)}\Gamma_{p}\left(\frac{1}{2}n\right)}{{\left(2\pi i\right)}^{\frac{1}{2}p\left(p+1\right)}}\int_{R(T)>0}e^{trT}{\left|T\right|}^{-\frac{1}{2}n}{}_{0}F_{0}\left(T^{-1}\Omega ,W\right)\left(dT\right). \end{array}$$

Without loss of generality, we may assume that $T=\mathrm{diag}(t_{1},\ldots ,t_{p})$ and also let $T^{-1}\Omega =U^{-1}$, then the Equation (16) becomes

(17) $$\begin{array}{c} {}_{0}F_{1}\left(b;\Omega ,W\right)\\ =\frac{2^{\frac{1}{2}p\left(p-1\right)}\Gamma_{p}\left(\frac{1}{2}n\right)}{{\left(2\pi i\right)}^{\frac{1}{2}p\left(p+1\right)}}\int_{R(U)>0}e^{tr(\Omega U)}{\left|\Omega U\right|}^{-\frac{1}{2}n}{}_{0}F_{0}\left(U^{-1},W\right){\left|\Omega \right|}^{p}\left(dU\right)\\ =\frac{2^{\frac{1}{2}p\left(p-1\right)}\Gamma_{p}\left(\frac{1}{2}n\right)}{{\left(2\pi i\right)}^{\frac{1}{2}p\left(p+1\right)}}\int_{R(U)>0}g\left(u_{1},\ldots ,u_{p};\Omega \right){}_{0}F_{0}\left(U^{-1},W\right)\left(dU\right), \end{array}$$

where

(18) $$\begin{array}{c} g\left(u_{1},\ldots ,u_{p};\Omega \right)=e^{\sum_{i=1}^{p}\omega_{i}u_{i}}\prod_{i=1}^{p}\omega_{i}^{-\frac{1}{2}n+p}\prod_{i=1}^{p}u_{i}^{-\frac{1}{2}n}. \end{array}$$

**Theorem** **5.** *${}_{0}F_{1}\left(\frac{1}{2}n;\Omega ,W\right)$ is $MTP_{2}$.*

**Proof.**  By Equation (18), after some straightforward calculations, we have

$$\begin{array}{c} \frac{\partial^{2}\log g\left(u_{1},\ldots ,u_{p};\Omega \right)}{\partial \omega_{i}\partial u_{i}}=1>0,\forall i=1,\ldots ,p. \end{array}$$

Thus, $g(u_{1},\ldots ,u_{p};\Omega )$ is $TP_{2}$ pairwise in $\left(u_{i},\delta_{i}\right)$. Namely, $g(u_{1},\ldots ,u_{p};\Omega )$ is $MTP_{2}$. By the composition lemma (Proposition 1) of Karlin [10], thus the theorem follows. □

Please note that $\psi_{0}\left(w_{1},\ldots ,w_{p}\right)$ is $MTP_{2}$, thus by Theorem 5, we may conclude that the density function $\psi_{\omega }\left(w_{1},\ldots ,w_{p};\Omega \right)$ is $MTP_{2}$.

### 4.4. Type ${}_{1}F_{1}$, Confluent Hypergeometric: Noncentral Eigenvalues

Suppose that the $p\times n_{1}$ matrix variate X with $n_{1}$ independent columns distributed as N(M,Σ) and $p\times n_{2}$ matrix variate Y with $n_{2}$ independent columns distributed as N(0,Σ) for $n_{1}\geq p$. Let $\omega_{i}^{*}$ be the eigenvalues of $|MM^{⊤}-\omega^{*}\Sigma |=0$. Write $\Omega^{*}=\mathrm{diag}\left(\omega_{1}^{*},\ldots ,\omega_{p}^{*}\right)$, then the joint density function of eigenvalues $f_{i}$ of $|XX^{⊤}-fYY^{⊤}|=0$, (i) for $n_{1}\geq p$, is of the form

(19) $$\begin{array}{c} \phi_{\omega^{*}}^{*}\left(f_{1},\ldots ,f_{p};\Omega^{*}\right)\\ =e^{-\frac{1}{2}\mathrm{tr}\Omega^{*}}{}_{1}F_{1}\left(\frac{1}{2}\left(n_{1}+n_{2}\right);\frac{1}{2}n_{2};\frac{1}{2}\Omega^{*},{\left(I+F^{-1}\right)}^{-1}\right)\phi^{*}\left(f_{1},\ldots ,f_{p}\right), \end{array}$$

where

(20) $$\begin{array}{c} \phi^{*}\left(f_{1},\ldots ,f_{p}\right)=\frac{\pi^{\frac{1}{2}p^{2}}\Gamma_{p}\left(\frac{1}{2}\left(n_{1}+n_{2}\right)\right)}{\Gamma_{p}\left(\frac{1}{2}n_{1}\right)\Gamma_{p}\left(\frac{1}{2}n_{2}\right)\Gamma_{p}\left(\frac{1}{2}p\right)}\frac{{\left|F\right|}^{\frac{1}{2}\left(n_{1}-p-1\right)}}{{\left|I+F\right|}^{\frac{1}{2}\left(n_{1}+n_{2}\right)}}\prod_{i<j}\left(f_{i}-f_{j}\right). \end{array}$$

**Theorem** **6.** ${}_{1}F_{1}\left(\frac{1}{2}n;\frac{1}{2}n_{2};\frac{1}{2}\Omega^{*},{\left(I+F^{-1}\right)}^{-1}\right)$ is $MRR_{2}$, where $n=n_{1}+n_{2}$.

**Proof.**  Please note that

$$\begin{array}{cc} {}_{1}F_{0}\left(\frac{1}{2}n;\Omega^{*-1},{\left(I+F^{-1}\right)}^{-1}\right) & =\int_{O(p)}{\left|I-\Omega^{*-1}H{\left(I+F^{-1}\right)}^{-1}H^{⊤}\right|}^{-\frac{1}{2}n}\left(dH\right)\\ & =|\Omega^{*}{|}^{\frac{1}{2}n}\int_{O(p)}{\left|\Omega^{*}-H{\left(I+F^{-1}\right)}^{-1}H^{⊤}\right|}^{-\frac{1}{2}n}\left(dH\right). \end{array}$$

Let $G={\left(I+F^{-1}\right)}^{-1}$ and $C(\Omega^{*},G)=\int_{O(p)}{\left|\Omega^{*}-HGH^{⊤}\right|}^{-n/2}\left(dH\right)>0$, then

$$\begin{array}{cc} & \frac{\partial^{2}\log{}_{1}F_{0}\left(\frac{1}{2}n;\Omega^{*-1},G\right)}{\partial \Omega^{*}\partial G}\\ & =-\{\frac{1}{4}n^{2}\int_{O(p)}|\Omega^{*}-HGH^{⊤}{|}^{-\frac{1}{2}n}{\left(\Omega^{*}-HGH^{⊤}\right)}^{-2}\left(dH\right)\times C\left(\Omega^{*},G\right)\\ & -\frac{1}{4}n^{2}[\int_{O(p)}|\Omega^{*}-HGH^{⊤}{{|}^{-\frac{1}{2}n}{\left(\Omega^{*}-HGH^{⊤}\right)}^{-1}\left(dH\right)]}^{2}\\ & +\frac{1}{2}n\int_{O(p)}|\Omega^{*}-HGH^{⊤}{|}^{-\frac{1}{2}n}{\left(\Omega^{*}-HH^{⊤}\right)}^{-2}\left(dH\right)\times C\left(\Omega^{*},G\right)\}/C^{2}\left(\Omega^{*},G\right). \end{array}$$

By Schwartz inequality, then

$$\begin{array}{cc} & \frac{\partial^{2}\log{}_{1}F_{0}\left(\frac{1}{2}n;\Omega^{*-1},G\right)}{\partial \Omega^{*}\partial G}\\ & \leq -\frac{1}{2}n\int_{O(p)}{\left|\Omega^{*}-HGH^{⊤}\right|}^{-\frac{1}{2}n}{\left(\Omega^{*}-HGH^{⊤}\right)}^{-2}\left(dH\right)/C\left(\Omega^{*},G\right)\\ & =-\frac{1}{2}nE_{g}\left[{\left(\Omega^{*}+HGH^{⊤}\right)}^{-2}|G;\Omega^{*}\right]\\ & \leq 0 \end{array}$$

with probability one, where $g\left(H|G;\Omega^{*}\right)={\left|\Omega +HGH^{⊤}\right|}^{-n/2}/C(\Omega^{*},G)$ can be viewed as the conditional density function of H given G. Please note that the matrix $HGH^{⊤}$ is positive definite with probability one, thus the conditional expectation $E_{g}\left[{\left(\Omega^{*}+HGH^{⊤}\right)}^{-2}|G;\Omega^{*}\right]$ is positive definite with probability one. Then, we have that ${}_{1}F_{0}\left(\frac{1}{2}n;\Omega^{*-1},G\right)$ is $MRR_{2}$ in $\left(\Omega^{*},G\right)$. Also, it is easy to note that G is the monotone increasing function of F, thus, ${}_{1}F_{0}\left(\frac{1}{2}n;\Omega^{*-1},{\left(I+F^{-1}\right)}^{-1}\right)$ is $MRR_{2}$ in $\left(\Omega^{*},F\right)$.

Next, consider

(21) $$\begin{array}{c} {}_{1}F_{1}\left(\frac{1}{2}n,\frac{1}{2}n_{2};\Omega^{*},{\left(I+F^{-1}\right)}^{-1}\right)\\ =\frac{2^{\frac{1}{2}p\left(p-1\right)}\Gamma_{p}\left(\frac{1}{2}n_{2}\right)}{{\left(2\pi i\right)}^{\frac{1}{2}p\left(p+1\right)}}\int_{R(T)>0}e^{trT}{\left|T\right|}^{-\frac{1}{2}n_{2}}{}_{1}F_{0}\left(\frac{1}{2}n;T^{-1}\Omega^{*},{\left(I+F^{-1}\right)}^{-1}\right)\left(dT\right). \end{array}$$

Let $T^{-1}\Omega^{*}=U^{-1}$, then

$$\begin{array}{cc} & {}_{1}F_{1}\left(\frac{1}{2}n,\frac{1}{2}n_{2};\Omega^{*},{\left(I+F^{-1}\right)}^{-1}\right)\\ & =\frac{2^{\frac{1}{2}p\left(p-1\right)}\Gamma_{p}\left(\frac{1}{2}n_{2}\right)}{{\left(2\pi i\right)}^{\frac{1}{2}p\left(p+1\right)}}\int_{R(U)>0}e^{tr(\Omega^{*}U)}|\Omega^{*}{U|}^{-\frac{1}{2}n_{2}}{}_{1}F_{0}\left(\frac{1}{2}n;U^{-1},W\right){\left|\Omega^{*}\right|}^{p}\left(dU\right)\\ & =\frac{2^{\frac{1}{2}p\left(p-1\right)}\Gamma_{p}\left(\frac{1}{2}n_{2}\right)}{{\left(2\pi i\right)}^{p(p+1)/2}}\int_{R(U)>0}g^{*}\left(u_{1},\ldots ,u_{p};\Omega^{*}\right){}_{1}F_{0}\left(\frac{1}{2}n;U^{-1},W\right)\left(dU\right), \end{array}$$

where

(22) $$\begin{array}{c} g^{*}\left(u_{1},\ldots ,u_{p};\Omega^{*}\right)=e^{\sum_{i=1}^{p}\omega_{i}^{*}u_{i}}\prod_{i=1}^{p}{\omega^{*}}_{i}^{-\frac{1}{2}n_{2}+p}\prod_{i=1}^{p}u_{i}^{-\frac{1}{2}n_{2}}. \end{array}$$

After some straightforward calculations, we have

$$\begin{array}{c} \frac{\partial^{2}\log g^{*}\left(u_{1},\ldots ,u_{p};\Omega^{*}\right)}{\partial \omega_{i}^{*}\partial u_{i}}=1>0,\;u_{i},\forall i=1,\ldots ,p. \end{array}$$

Thus, $g^{*}\left(u_{1},\ldots ,u_{p};\Omega^{*}\right)$ is $TP_{2}$ pairwise in $\left(u_{i},\omega_{i}^{*}\right)$. Namely, $g^{*}\left(u_{1},\ldots ,u_{p};\Omega^{*}\right)$ is $MTP_{2}$. By the composition lemma (Proposition 1) of Karlin [10], then the theorem follows. □

Similar to arguments in Example 5, we may have that $\phi^{*}\left(f_{1},\ldots ,f_{p}\right)$ is $MRR_{2}$. Thus by Theorem 6, we may conclude that the density function $\phi_{\omega^{*}}^{*}\left(f_{1},\ldots ,f_{p};\Omega^{*}\right)$, which is the product of two $MRR_{2}$ functions (Karlin and Rinott [13]), is $MRR_{2}$.

(ii) For $p\geq n_{1}$ the joint density function of eigenvalues $f_{1},\ldots ,f_{p}$ is of the form

(23) $$\begin{array}{c} \phi_{2\omega^{*}}^{*}\left(f_{1},\ldots ,f_{p};\Omega^{*}\right)\\ =e^{-\frac{1}{2}\mathrm{tr}\Omega^{*}}{}_{1}F_{1}\left(\frac{1}{2}\left(n_{1}+n_{2}\right);\frac{1}{2}p;\frac{1}{2}\Omega^{*},{\left(I+F^{-1}\right)}^{-1}\right)\phi_{2}^{*}\left(f_{1},\ldots ,f_{p}\right), \end{array}$$

where

(24) $$\begin{array}{c} \phi_{2}^{*}\left(f_{1},\ldots ,f_{p}\right)\\ =\frac{\pi^{\frac{1}{2}n_{1}^{2}}\Gamma_{n_{1}}\left(\frac{1}{2}\left(n_{1}+n_{2}\right)\right)}{\Gamma_{n_{1}}\left(\frac{1}{2}n_{1}\right)\Gamma_{n_{1}}\left(\frac{1}{2}\left(n_{1}+n_{2}-p\right)\right)\Gamma_{n_{1}}\left(\frac{1}{2}p\right)}\frac{{\left|F\right|}^{\frac{1}{2}\left(p-n_{1}-1\right)}}{{\left|I+F\right|}^{\frac{1}{2}\left(n_{1}+n_{2}\right)}}\prod_{i<j}\left(f_{i}-f_{j}\right). \end{array}$$

Similar to arguments in Theorem 6, we may conclude that the density function $\phi_{2\omega^{*}}^{*}\left(f_{1},\ldots ,f_{p};\Omega^{*}\right)$ is $MRR_{2}$.

### 4.5. High-Dimensional Wishart Matrices

Suppose that the columns of a matrix p×n matrix X are independently normally distributed with covariance matrix Σ and E(X)=0. Let $L=\mathrm{diag}(l_{1},\ldots ,l_{p})$, where $l_{i}$ are the *i*th largest eigenvalue of $XX^{⊤}$. Let $\Sigma =\sigma^{2}I$ and $c=\lim_{n\to \infty }p/n,c\in \left(0,1\right)$. When dimension *p* is large, the limiting distribution of sample spectral eigenvalues has the well-known Mar$\overset{ˇ}{c}$enko-Pastur distribution $F_{c,\sigma^{2}}$ (M-P law) with index *c* and scale parameter σ, which the density function is of the form

$$\begin{array}{c} f_{c,\sigma^{2}}\left(x\right)=\frac{1}{2\pi xc\sigma^{2}}\sqrt{\left(b\sigma^{2}-x\right)\left(x-a\sigma^{2}\right)},\;a\sigma^{2}\leq x\leq b\sigma^{2}, \end{array}$$

where $a={\left(1-\sqrt{c}\right)}^{2}$ and $b={\left(1+\sqrt{c}\right)}^{2}$ (Mar$\overset{ˇ}{c}$enko and Paster [21]). After some algebraic manipulations, we can show that

$$\begin{array}{cc} \frac{\partial^{2}\log f_{c,\sigma^{2}}\left(x\right)}{\partial x\partial \sigma^{2}} & =\frac{1}{4\pi c}\frac{\left(a+b\right)x^{2}-4abx\sigma^{2}+\left(a+b\right)ab\sigma^{4}}{{\left(b\sigma^{2}-x\right)}^{2}{\left(x-a\sigma^{2}\right)}^{2}}\\ & \geq 0. \end{array}$$

Thus, the Mar$\overset{ˇ}{c}$enko-Pastur density function is $TP_{2}$ in $\left(x,\sigma^{2}\right)$, and hence Theorem 1 is applicable.

## 5. Remarks

We adopted the notion of convex ordering of two distributions to prove the ordering of Shannon entropies in Theorem 2. Please note that the notions of convex ordering of two distributions, the monotone likelihood ratio and totally positive of order 2 ($MTP_{2}$) are essentially equivalent. For many density functions such as those discussed in Section 3 and Section 4, it seems easier to characterize the $MTP_{2}$ property of density functions than that of convex ordering of distribution functions. In practice, we suggest use the notion of $MTP_{2}$ for the comparisons of Shannon entropy measures.

The difference of two Shannon entropies is an intrinsic distribution measure as we emphasized in this paper. As a result, the Shannon entropy measures can be ordered when two underline distributions have the relationship of convex ordering (i.e., the $MTP_{2}$) property. The monotonicity of power function, which is discussed basically under the same distributions but with different ordered parameters set up, comes out to be a special case of the results of the comparison of two Shannon entropies which can be even discussed under two totally different distribution functions.

For the problem of hypothesis testing $H_{0}:F\left(x\right){=}_{c}G\left(x\right)$ against $H_{1}:F\left(x\right)\leq_{c}G\left(x\right)$, test statistic based on the sample version of the difference of two Shannon entropies enjoys the optimal property. The above hypothesis testing problem is equivalent to the hypothesis problem of testing $H_{0}:H_{F}\left(u\right)=u,u\in \left[0,1\right]$ against $H_{1}:H_{F}\left(u\right)$ is convex on [0,1]. Let $U_{1:n}<U_{2:n}<\ldots <U_{n:n}$ be the order statistics from $H_{F}$, then the most powerful test is based on the statistics $\sum_{i=1}^{n}log\;h_{F}\left(U_{i:n}\right)$, which is the empirical version of the difference of two Shannon entropies. Since $h_{F}\left(u\right)$ is monotone increasing in *u*, thus the test has the property of power monotonicity.

Noncentral chi-square distributions play an important role for statistical inference. For the univariate case, a noncentral chi-square or gamma density which are typically Poisson mixture of central chi-square or gamma type densities. Beyond the monotonicity property, we may aware of the following: let $f_{\alpha }\left(x,\beta \right)$ denote the univariate gamma density function with the shape parameter α and the scale parameter β, and its distribution function is denoted by $F_{\alpha }\left(x,\beta \right)$. For the fixed scale parameter gamma densities, van Zwet [6] showed the result of convex ordering of gamma distributions, i.e., if $0<\alpha_{1}\leq \alpha_{2}$ then $F_{\alpha_{2}}\left(x,\beta \right)\leq_{c}F_{\alpha_{1}}\left(x,\beta \right)$. Namely, $f_{\alpha }\left(x,\beta \right)$ is $TP_{2}$ in (x,α) when β is fixed. Thus, by Theorem 1 if $0<\alpha_{1}\leq \alpha_{2}$ then $I_{S}\left(f_{\alpha_{1}}\right)\leq I_{S}\left(f_{\alpha_{2}}\right)$ when β is fixed. Similarly, when α is fixed $f_{\alpha }\left(x,\beta \right)$ is $TP_{2}$ in (x,β). The above results tell us that the larger degrees of freedom is, the larger Shannon entropy measure is when both noncentralities are kept the same. For any two test statistics (or estimators) which are noncentral chi-square distributed with the same noncentrality, but with different degrees of freedom, then the Pitman efficiency (based on the Kullback-Leibler divergence) makes no difference of comparisons. However, our new measure of the difference of two Shannon entropies is nonzero, which means that the new measure makes a difference of comparisons. For the MANOVA models, the density functions of eigenvalues which are Bessel of multivariate gamma type density or confluent hypergeometric of multivariate beta type density such as those discussed in Section 4.3 and Section 4.4, respectively, our results can be directly applied to the tests with monotone acceptance regions to enjoy the power monotonicity for those density functions with the $MTP_{2}$ (MLR) property.

The conceived mixture model arises also for the finite case. A multi-sample model where for some G(G>1), there are *G* densities $f_{g}\left(x\right),g=1,\ldots ,G$ and a sample of size $n_{g}$ is drawn from the density $f_{g}\left(x\right)$, so that the pooled sample relates to the mixture density with $w_{g}=n_{g}/\sum_{g=1}^{G}n_{g}$. For the finite mixture case, it is hard to find out the explicit form of Shannon entropy of mixture distribution, even under the multinormal set-up. Suppose that there are *G* groups, the random vector X has the mixture density function $f_{G}^{*}\left(x\right)=\sum_{g=1}^{G}w_{g}f_{g}\left(x\right)$, where $\sum_{g=1}^{G}w_{g}=1$. Then the distribution function is $F_{G}^{*}\left(x\right)=P\left\{X\leq x\right\}=\int_{-\infty }^{x}\sum_{g=1}^{G}w_{g}f_{g}\left(y\right)dy=\sum_{g=1}^{G}w_{g}\int_{-\infty }^{x}f_{g}\left(y\right)dy=\sum_{g=1}^{G}w_{g}F_{g}\left(x\right)$. The $w_{g}$ are nonnegative and they add up to 1, so the mixture density represents a convex combination of the component densities. We find that the decomposability of Shannon entropy still holds in a different way for the continuous random vectors.

**Theorem** **7.** $I_{S}\left(f_{G}^{*}\right)=\sum_{g=1}^{G}w_{g}I_{S}\left(f_{g}\right)+\sum_{g=1}^{G}w_{g}K\left(f_{g},f_{G}^{*}\right)$.

**Proof.**  Please note that

$$\begin{array}{cc} I_{S}\left(f_{G}^{*}\right) & =\int_{-\infty }^{\infty }-\log f_{G}^{*}\left(x\right)dF_{G}^{*}\left(x\right)\\ & =-\int_{-\infty }^{\infty }\log\left(\sum_{g=1}^{G}w_{g}f_{g}\left(x\right)\right)d\left[\sum_{i=1}^{G}w_{i}F_{i}\left(x\right)\right]\\ & =-\sum_{g=1}^{G}w_{g}\int_{-\infty }^{\infty }f_{g}\left(x\right)\log\left(\sum_{i=1}^{G}w_{i}f_{i}\left(x\right)\right)dx\\ & =-\sum_{g=1}^{G}w_{g}\int_{-\infty }^{\infty }f_{g}\left(x\right)\log f_{g}\left(x\right)dx+\sum_{g=1}^{G}w_{g}\int_{-\infty }^{\infty }f_{g}\left(x\right)\log\left(\frac{f_{g}\left(x\right)}{\sum_{i=1}^{G}w_{i}f_{i}\left(x\right)}\right)dx\\ & =\sum_{g=1}^{G}w_{g}I_{S}\left(f_{g}\right)+\sum_{g=1}^{G}w_{g}K\left(f_{g},f_{G}^{*}\right). \end{array}$$

 □

It follows from Theorem 7 that $I_{S}\left(f_{G}^{*}\right)\geq \sum_{g=1}^{G}w_{g}I_{S}\left(f_{g}\right)$. We further note that for a mixture model, the Shannon entropy has the decomposability property similar to well-known Fisher’s MANOVA model. Please note that the first term on the right hand side represents an average of the individual entropies (i.e., analogous to the within group sum of squares) while the second term is nonnegative and represents the between group distances.

For a high-dimensional Wishart matrix with an unknown scale times the identity matrix being the population covariance matrix, the property of $TP_{2}$ property (i.e., the MLR property) for the limiting empirical density function of eigenvalues is studied in the Section 4.5. Although the Mar$\overset{ˇ}{c}$enko-Pastur equation (Silverstein [22]) provides the link of limiting empirical spectral distribution, *F*, to the limiting behavior of the population spectral distribution, *H*, one can expect to retrieve the information of *H* from *F*. However, the difficulty lies within the fact that the relationship between *H* and *F* is entangled. Whether the result studied in Section 4.5 can be extended to the general population covariance matrix case or not remains to be clarified. Another focus of random matrices in the literature is related to the Gaussian divisible ensembles. The Gaussian divisible ensembles are matrices of the form $H_{t}=e^{-t/2}H_{0}+{\left(1-e^{-t}\right)}^{1/2}H_{G}$ where t>0 is a parameter, $H_{0}$ is a Wigner matrix and $H_{G}$ is an independent Gaussian orthogonal ensemble matrix. The eigenvalue distribution of the Gaussian divisible ensembles are the same as that of the solution of a matrix valued Ornstein-Uhlenbeck process $H_{t}$ for any time t≥0. The dynamics of the eigenvalues of $H_{t}$ is given by the system of so-called Dyson’s Brownian motion (Dyson [23]). The treatment of the sample covariance matrix is analogous, but the formulas change slightly (see Erd$\overset{ʺ}{o}$s, L. et al. [24], for the details). It is interesting to further study that: under the general model, whether the corresponding limiting spectral density function of eigenvalues, obtained via the convolution of the limiting density function of eigenvalues of initial matrix with the Mar$\overset{ˇ}{c}$enko-Pastur density function, is $TP_{2}$ or not. We pose those problems as a project of future study.
